# Plumes of neuronal activity propagate in three dimensions through the nuclear avian brain

**DOI:** 10.1186/1741-7007-12-16

**Published:** 2014-02-28

**Authors:** Gabriël JL Beckers, Jacqueline van der Meij, John A Lesku, Niels C Rattenborg

**Affiliations:** 1 Avian Sleep Group, Max Planck Institute for Ornithology, Eberhard-Gwinner-Strasse 11, 82319 Seewiesen, Germany; 2 Cognitive Neurobiology and Helmholtz Institute, Departments of Psychology and Biology, Utrecht University, PO Box 80086, 3508 TB Utrecht, The Netherlands; 3 Department of Zoology, La Trobe University, Kingsbury Drive, Melbourne VIC 3086, Australia

**Keywords:** Sleep, Slow waves, Propagation, Travelling, Bird, Cortex

## Abstract

**Background:**

In mammals, the slow-oscillations of neuronal membrane potentials (reflected in the electroencephalogram as high-amplitude, slow-waves), which occur during non-rapid eye movement sleep and anesthesia, propagate across the neocortex largely as two-dimensional traveling waves. However, it remains unknown if the traveling nature of slow-waves is unique to the laminar cytoarchitecture and associated computational properties of the neocortex.

**Results:**

We demonstrate that local field potential slow-waves and correlated multiunit activity propagate as complex three-dimensional plumes of neuronal activity through the avian brain, owing to its non-laminar, nuclear neuronal cytoarchitecture.

**Conclusions:**

The traveling nature of slow-waves is not dependent upon the laminar organization of the neocortex, and is unlikely to subserve functions unique to this pattern of neuronal organization. Finally, the three-dimensional geometry of propagating plumes may reflect computational properties not found in mammals that contributed to the evolution of nuclear neuronal organization and complex cognition in birds.

## Background

Historically, the mammalian neocortex has been viewed as the pinnacle of brain evolution. The highly structured six-layered laminar cytoarchitecture of the neocortex and the associated computational properties contributing to complex cognition added to this view. However, exactly how laminar cytoarchitecture and associated neurophysiological processes mediate complex cognition remains poorly understood. Our understanding of how the neocortex works can be informed by comparisons with other animals. Notably, comparisons with non-mammalian groups lacking laminar cytoarchitecture, as is the case in birds, can be used to isolate traits that depend upon laminar cytoarchitecture from those that do not. In this study, we use this comparative approach to gain insight into the neocortex by examining sleep-related neuronal activity in the avian brain.

A growing body of research suggests that the brain rhythms occurring during sleep are involved in processing information acquired during wakefulness [1]. Notably, the slow (typically <1 Hz) oscillation of neocortical neuronal membrane potentials between a depolarized “up-state” with action potentials, and a hyperpolarized “down-state” with neuronal quiescence occurring during non-rapid eye movement (NREM) sleep and some types of anesthesia is the focus of several hypotheses for the function of sleep [2-4]. The term ‘slow-oscillation’ is in wide-spread use even though the interval between down-states is variable and individual cycles of the slow-oscillation originate from different neocortical locations [5-8]. Slow-oscillations manifest in electroencephalogram (EEG) or local field potential (LFP) recordings as high-amplitude, slow-waves that propagate across the mammalian neocortex as traveling waves [5,7-15], raising the possibility that they are involved in processing spatially distributed information [1,16] via processes such as spike timing-dependent plasticity [17]. However, it remains unknown whether the traveling nature of slow-oscillations reflects a feature unique to the laminar cytoarchitecture and associated computational properties of the neocortex [18] or a more general aspect of sleep-related neuronal activity.

To distinguish between these alternatives, we studied brain activity in birds, the only non-mammalian group known to exhibit slow-oscillations [19] and associated EEG slow-waves comparable to those observed in mammals during NREM sleep [20,21]. This similarity between mammals and birds is particularly interesting because unlike the laminar mammalian neocortex, neurons in the avian forebrain are arranged in a largely nuclear manner [22]. Specifically, the hyperpallium, a region developmentally homologous and functionally similar to the mammalian primary visual and somatosensory/motor cortices [23,24], lacks the laminar arrangement of neurons, including pyramidal neurons with long trans-layer apical dendrites found in the six-layered mammalian neocortex and in the three-layered dorsal cortex of the closest living reptilian relatives to birds [24]. Instead, the hyperpallium is composed of long flat nuclei stacked one on top of the other running along the dorsal-medial-anterior surface of the brain, each of which is composed of stellate neurons with short spiny dendrites and axonal projections within and between nuclei [24,25]. Interestingly, this cytoarchitecture is even found within high-order association regions in the avian brain (that is, mesopallium and nidopallium) involved in orchestrating complex cognitive tasks, in some cases comparable to those performed by primates [26].

We recorded intracerebral potentials in the zebra finch (*Taeniopygia guttata*) hyperpallium and nidopallium to evaluate whether traveling slow-waves are unique to mammals, or are shared with birds irrespective of fundamental differences in cytoarchitectonic organization.

## Results and discussion

**Figure 1:**
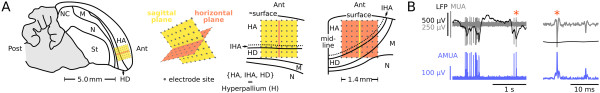
**High-density recording methods. (A)** Location of the 64-channel silicon multi-electrode probe. An 8 × 8 electrode grid with 200 μm inter-site spacing extends over a 1,400 × 1,400 μm plane in the hyperpallium. Probes were inserted horizontally (red plane) or sagitally (yellow plane). The horizontal plane is orthogonal to the sagittal plane and is shown as a thin line in the sagittal brain section. The orientation of horizontal electrode grids is always depicted so that the medial side is on the left and the anterior side (that is, brain surface) is on top; sagittal grids are depicted so that the caudal side is on the left and the anterior side (that is, brain surface) is on top. Together, horizontal and sagittal recordings provide insight into the three-dimensional spatial extent of activity propagation patterns. Thalamic input to the hyperpallium (H), which is where most recordings were performed (*n* = 11 birds), terminates primarily in the interstitial part of hyperpallium apicale (IHA), a thin region underlying the rest of hyperpallium apicale (HA) and overlying the hyperpallium densocellulare (HD). Output projections from the HA course medially over the surface of the brain before descending down the septomesencephalic tract. The hyperpallium overlies and is interconnected with the mesopallium (M) and nidopallium (N), large nuclear structures composed of similar stellate neurons, involved in high-order cognitive processes comparable to those performed by the neocortex [26]. Two additional recordings were performed in caudal nidopallium (NC; *n* = 2 birds). **(B)** Raw intracerebral signals were recorded and off-line filtered to a signal containing local field potentials (LFP, 0.1 to 350 Hz), and to a signal containing multiunit action potentials (MUA, 0.5 to 5 kHz). To obtain a signal that corresponds to the level of multiunit action potential firing, the MUA signal was rectified and decimated (analogue multiunit activity, AMUA).

**Figure 2:**
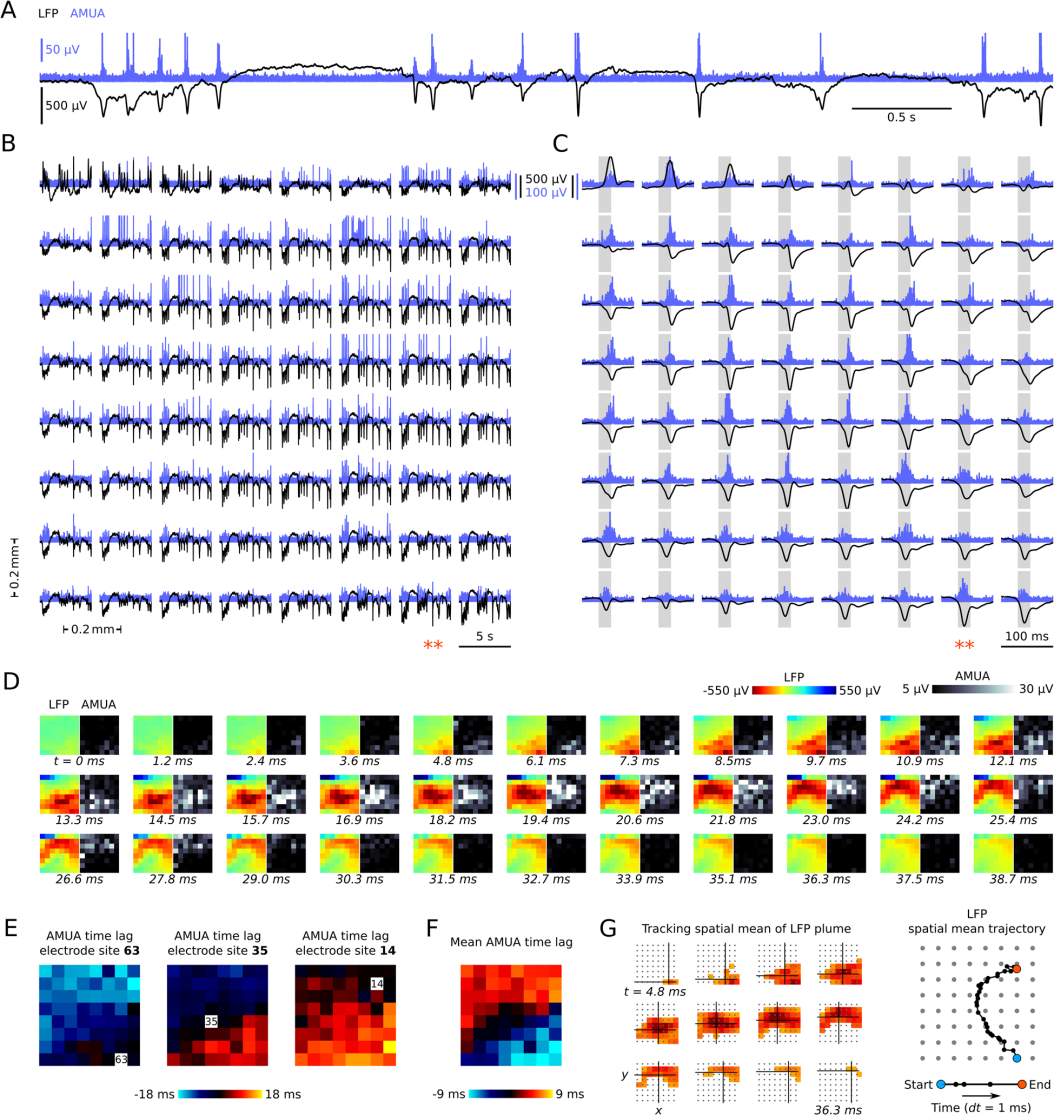
**Propagating slow-waves in the zebra finch forebrain. (A)** This is an expanded five-second recording showing the temporal pattern of local field potential (LFP) and analogue multiunit activity (AMUA) (Bird 6, horizontal plane, and electrode site column 6, row 4 from Figure 2B). **(B)** The same five-second example as in Figure 2A as recorded across the 8 × 8 grid of electrode sites, showing that oscillations appear to be globally distributed. **(C)** Detail of the LFP and AMUA peak indicated with two asterisks in Figure 2B, showing that peak activity occurs at slightly different times in different sites. **(D)** This is the same peak event as in Figure 2C, now visualized in a sequence of image plots, where each image has 8 × 8 pixels corresponding to the 8 × 8 grid of electrode sites, and where pixel color and gray levels correspond to LFP and AMUA magnitudes. The peak of the waveform plots in Figure 2C now appears as a propagating plume of activity. **(E)** The AMUA time lag of peak activity for three sites, each relative to the other sites, expressed in an 8 × 8 matrix of image pixels that correspond to the site positions in the electrode grid. Time lags are calculated as the lag of maximum cross-correlation, with cross-correlation lags ranging from −50 to +50 ms. See text for more explanation. **(F)** The mean lag of an electrode site relative to the other sites (mean of *n* = 63). Each pixel is the mean of the values as shown in an 8 × 8 matrix in Figure 2E. **(G)** LFP plume propagation across the grid is tracked by calculating the spatial average of the plume in 1-ms intervals, based on sites with a potential < −0.25 mV. Cross hairs indicate plume spatial average for a selection of 12 1-ms time intervals.

Analogue multiunit activity (AMUA) and LFP were recorded using a 64-channel silicon multi-electrode probe inserted into the hyperpallium (Figure 1A, B) and caudal nidopallium of adult female zebra finches anesthetized with 1 to 1.25% isoflurane [27,28], an anesthetic that activates normal sleep promoting regions in the mammalian [29] and fly [30] brain. Except in a thin layer just below the surface of the brain, hyperpallial LFP oscillations consist of negatively peaked signals (Figure 2A, B). In contrast, the corresponding potentials near the surface of the brain are often positively peaked (Figure 2B-D). Similar intra-hyperpallial phase reversals have also been observed in visually evoked potentials in anesthetized zebra finches [31]. Unlike the mammalian neocortex, wherein long apical dendrites extending toward the surface of the brain are thought to give rise to similar phase reversals [32], there are no known neuronal asymmetries (that is, apical dendrites) that might cause these positively peaked potentials in finches, other than output axons projecting across the surface of the hyperpallium to the septomesencephalic tract [25]. Nonetheless, these oscillations are likely the basis for slow-waves recorded from epidural EEG electrodes during NREM sleep in finches and other birds [33].

At a time scale of seconds, LFP patterns appear visually similar between most electrode sites (Figure 2B), even distant ones, and are strongly correlated (for negatively-peaked sites mean between-site correlation coefficients range from 0.72 to 0.92; median 0.84; *n* = 11 birds), giving the impression that oscillations are near-synchronous across brain regions and thus globally organized. As is the case in the mammalian neocortex [34,35], at most of these sites negative LFP peaks coincide with bursts of action potential activity (mean correlation coefficients between LFP and AMUA signals range from 0.38 to 0.60; median 0.50, *n* = 11 birds). Sites with positive LFP peaks near the surface are less likely to be associated with strong action potential firing in the same recording site (Additional file 1: Figure S1).

At shorter time-scales of tens to hundreds of milliseconds, however, single oscillations do not appear as near-synchronous and globally distributed. For example, Figure 2C zooms in on a single LFP peak from Figure 2B, and shows that the peak occurs at slightly different times across the electrode grid, such that it occurs first at sites in the bottom right corner and then later at sites in the top half of the grid. Although it is possible to see such shifting of activity peaks in traditional waveform plots, the temporospatial dynamics are better visualized in sequences of image plots, especially when viewed as videos (see Additional file 2: Video S1, Additional file 3: Video S2, Additional file 4: Video S3, Additional file 5: Video S4, Additional file 6: Video S5, Additional file 7: Video S6, Additional file 8: Video S7, Additional file 9: Video S8, Additional file 10: Video S9). Figure 2D shows in a sequence of image plots that the traveling “peak” event in Figure 2C is a spatially localized plume of field and action potentials that propagates fast across the electrode grid (Additional file 2: Video S1). The fact that the LFP plume co-occurs with action potential firing (AMUA) suggests that it reflects local neurophysiological activity, rather than activity volume-conducted from a remote source.

**Figure 3:**
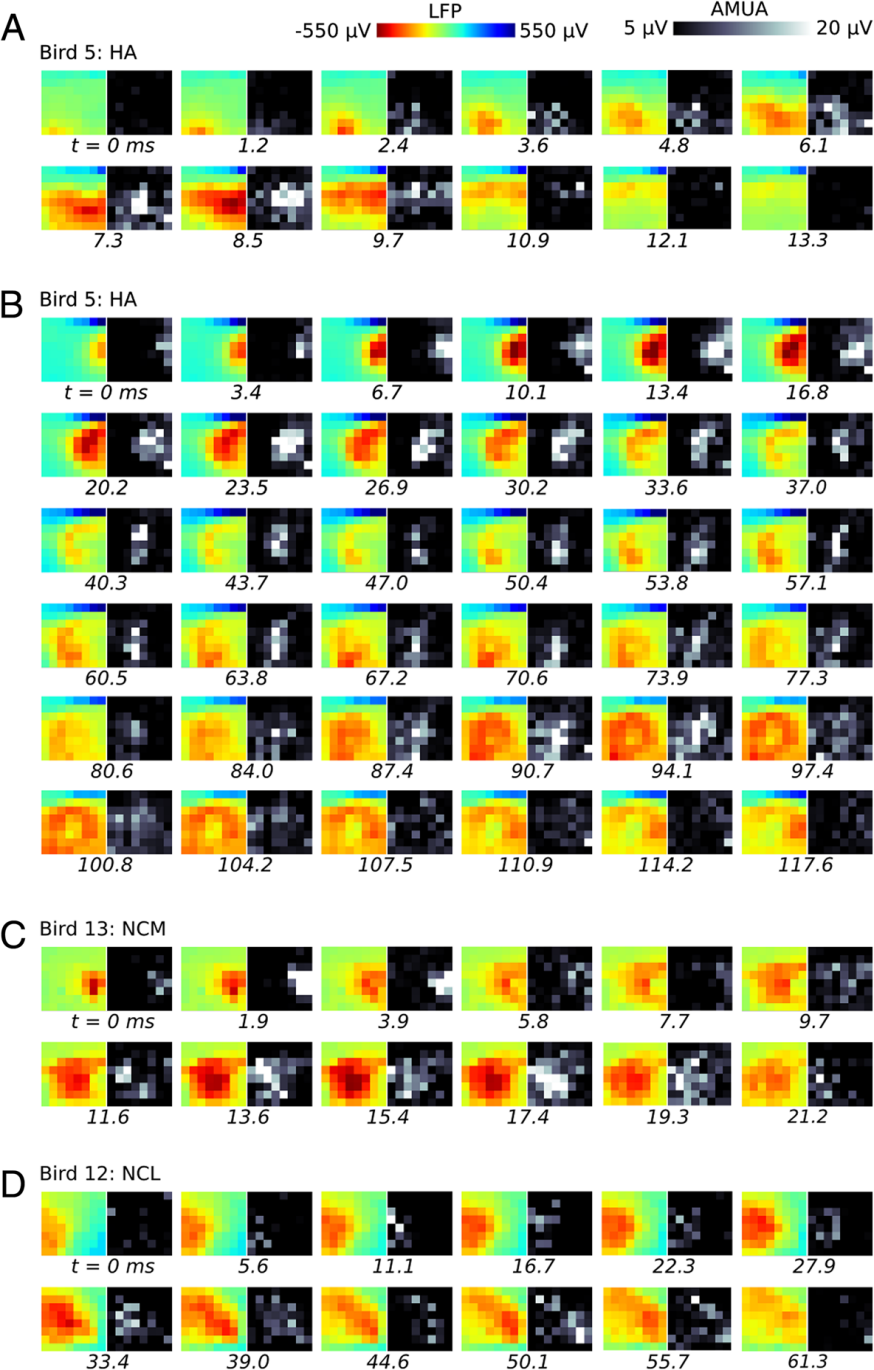
**Examples of propagating plumes. (A)** A plume in the hyperpallium of Bird 5 that sweeps across the grid in a similar direction as the plume in Figure 2 from Bird 6, but in a shorter time span. **(B)** In the same recording of Bird 5, plumes can propagate in different directions, and in complex ways. **(C)** A propagating plume in caudomedial nidopallium (NCM), a forebrain region that is distant from the hyperpallium, where most recordings in this study were performed. **(D)** A propagating plume in caudolateral nidopallium (NCL), a forebrain region that is maximally distant from the hyperpallium. This plume was recorded 3.5 mm lateral from that in NCM (in different birds).

We compiled videos from all of our recordings from the hyperpallium (*n* = 11 birds), and all of them show propagating local plumes of local field and action potential activity, such as seen in Figure 2D. Nonetheless, dynamic patterns of activity are generally variable across different plumes, even within the same bird. Figure 3A (Additional file 3: Video S2) shows in a different bird a plume that propagates to the surface of the brain, but in the same recording plumes can be seen propagating in different directions: a complex example is provided in Figure 3B (Additional file 4: Video S3), where a plume of activity propagates in a right (lateral) to left (medial) direction before briefly dissipating. The activity then strengthens again near the lower center of the array and expands outward as a circle of activity. Propagation of plumes is not confined within a two-dimensional layer, as they can travel over considerable distances in variable directions, including diametrically opposite ones, both within orthogonally placed electrode grids (horizontal and sagittal planes), and relative to the plane of the stacked hyperpallial nuclei. As can be seen in the movies (Additional file 2: Video S1, Additional file 3: Video S2, Additional file 4: Video S3, Additional file 5: Video S4, Additional file 6: Video S5, Additional file 7: Video S6, Additional file 8: Video S7, Additional file 9: Video S8, and Additional file 10: Video S9) and Additional file 11: Figure S2, plume propagation velocity across the 2-D electrode grid is variable. However, given that plumes have complex changing shapes and their 3-D trajectories may impinge upon the 2-D electrode grid at variable angles, it is not generally possible to estimate plume propagation velocity from translation velocity across the grid. Thus, whether or not plume propagation velocity is variable remains an open question. Furthermore, it is unclear how the propagation speed of avian plumes compares with that of two-dimensional slow-waves in the mammalian neocortex [5,8,13].

Although we focus on the hyperpallium in the current study, we wondered whether propagating plume activity occurs outside the hyperpallium. To address this question, we added two recordings (*n* = 2 birds) to our dataset, one in caudolateral nidopallium (NCL) and the other one in caudomedial nidopallium (NCM), which are forebrain regions that are maximally distant from the hyperpallium (Figure 1A). As in the hyperpallium, both recordings consist of prominent propagating plumes of local field and action potential activity (examples Figure 3C, D; Additional file 5: Videos S4 and Additional file 6: Video S5), suggesting that this is a general feature of the avian pallium.

Despite the large variability in propagation patterns that we observed in the videos of all recordings (the Additional file 7: Video S6, Additional file 8: Video S7, Additional file 9: Video S8, Additional file 10: Video S9 provide representative examples), there appear to be some recurrent patterns within recordings and a bias in overall directionality. Although the full complexity of plumes is not yet understood, and probably is too high to be meaningfully expressed in a low-dimensional set of statistics, we reasoned that non-randomness of propagation patterns may be revealed by investigating two directionality characteristics of plumes.

**Figure 4:**
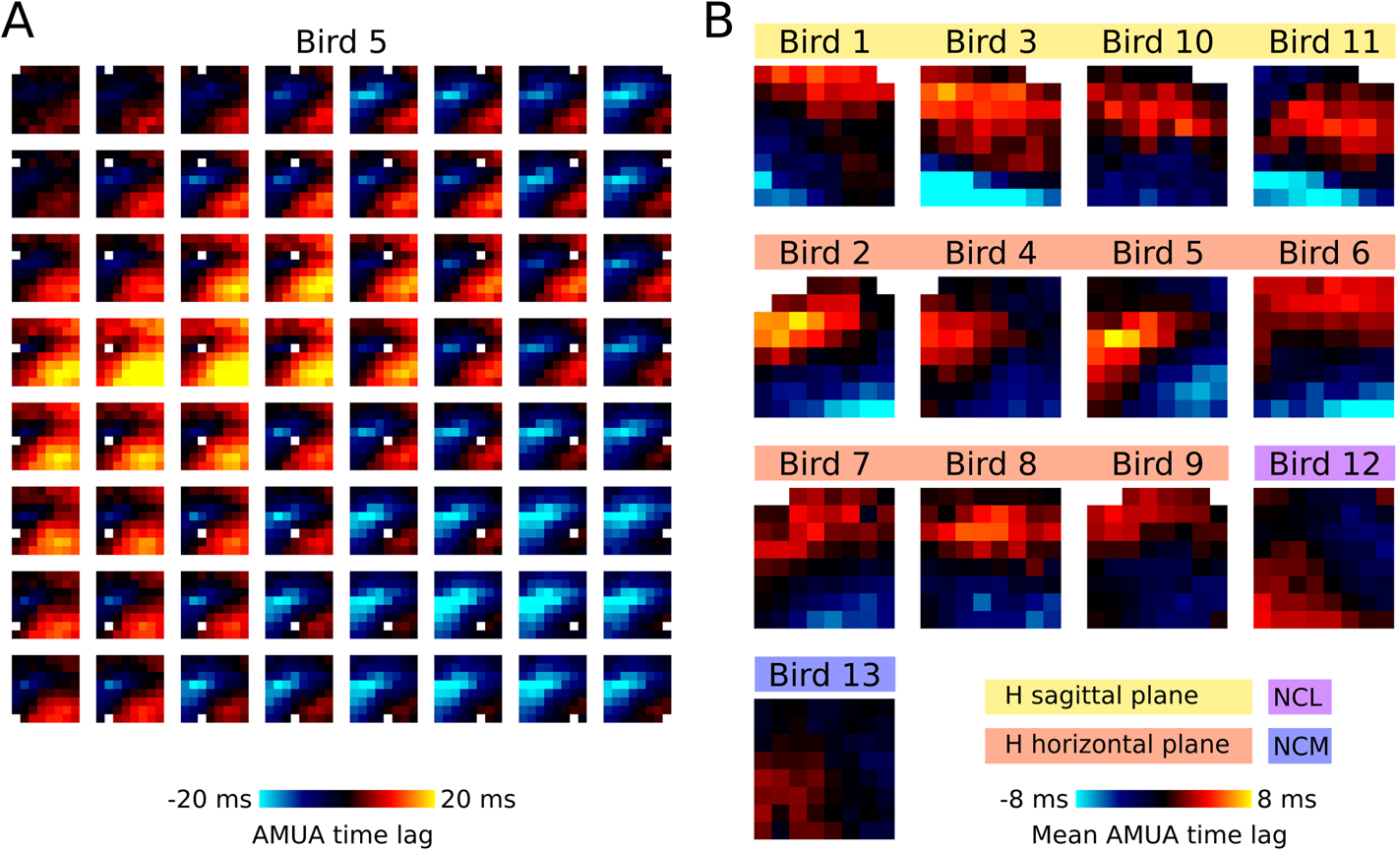
**Directional structure in action potential propagation.** Time lags of maximum between-electrode site cross-correlation of analogue multiunit activity (AMUA) signals show that despite the diversity of patterns in action potential activity propagation visible in the recording videos, there is directional structure in action potential plume propagation patterns. **(A)** This is an example of mean between-electrode time lags in the recording of Bird 5 (horizontal recording in the hyperpallium, H). Means are calculated over identified plumes (*n* = 712 plumes in Bird 5). Each subplot in the 8 × 8 matrix of image plots corresponds to an electrode site with the matrix arrangement of subplots corresponding to the locations of electrodes on the multi-electrode probe; cf. Figure 1A. Subplots are images in which the color of pixels represents the lag in milliseconds of the maximum cross-correlation between that particular site and each of the 63 other electrode sites (see Figure 2E and main text). **(B)** For every bird, the mean time lag of each electrode site with all other sites is shown (cf. Figure 2F).

First, to identify systematic time delays in AMUA activity between each electrode site and all other electrode sites, we calculated the maxima of normalized cross-correlation functions for each identified plume in every recording. Illustrating this process for the propagating plume shown in Figure 2C, D, Figure 2E visualizes for three electrode sites the lags of AMUA peak activity at those sites relative to all other sites, which are color-coded in pixels arranged according to the spatial distribution of sites in the electrode grid. AMUA at site 63 is leading with respect to the other sites, such that the maximum cross-correlation is achieved later, overall, as the distance to other electrode sites increases. The lag of site 35 is intermediate relative to that of the other sites, being delayed with respect to sites in the lower right quadrant, but leading with respect to sites in the upper left quadrant of the grid. Site 14 is delayed with respect to most other sites, maximally so relative to sites in the lower right quadrant. The information in each of these image plots is then averaged to a single value that expresses the mean lag of the corresponding site relative to other sites, and these mean values are visualized in a new image plot (Figure 2F). Note that the spatial distribution of site activity lag is consistent with the direction of the AMUA plume propagation visible in Figure 2D. To determine whether or not there is a systematic directionality in plume propagation overall, we calculated electrode site lag matrices for all identified plumes in all recordings, and averaged the result over plumes within recordings (10 minutes duration). An example is given for Bird 5 in Figure 4A, which shows that there is underlying structure in propagation dynamics, with plume action potential activity propagating more often from the bottom-right (posterior-lateral) corner of the grid to the top-left (anterior-medial) corner. The smooth distribution of time lags over space is consistent with the traveling propagation dynamics. Mean lag maps of recordings in the other birds (Figure 4B) show that in the hyperpallium activity of deeper (posterior) recording sites tends to lead with respect to anterior sites toward the surface of the brain, even though there is variability between birds in the exact spatial distribution of time lags. As such, at least some hyperpallial plumes may originate from the underlying mesopallium and/or nidopallium. Finally, as described for propagating slow-waves in the mammalian neocortex [9,15], in general terms, the overall directional bias in hyperpallial plume propagation is parallel to the predominant orientation of myelinated axons in the zebra finch hyperpallium [36]; although, as noted above, individual plumes often propagate orthogonal to this axonal orientation.

**Figure 5:**
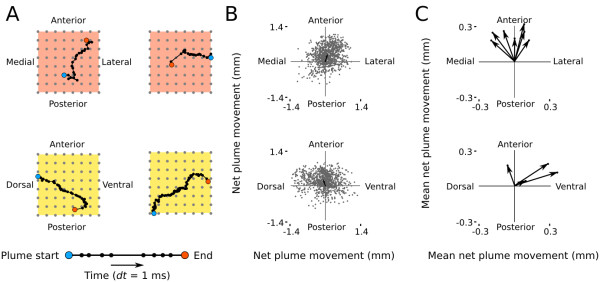
**Traveling LFP plume trajectories.** LFP plume mean location was determined in 1-ms time steps (see Experimental Procedures and Figure 2G). **(A)** LFP plumes can originate in different locations and travel along variable trajectories. Two examples from the same recording are given for each of the two orthogonal recording planes in the hyperpallium: horizontal (red) and sagittal (yellow; multi-electrode orientations are the same as in Figure 1A). **(B)** The net plume propagation along the two orthogonal axes of the multi-electrode array was calculated for every LFP plume in a recording; shown are two recordings from different birds. Every gray dot corresponds to one plume. Plume propagation is variable, but not completely random. The mean net LFP plume movement is indicated as a black arrow. **(C)** Mean net LFP peak movement vectors in the hyperpallium (one recording per bird; *n* = 11) illustrating the non-random nature of slow-wave propagation: plumes tend to propagate toward the surface of the brain.

Because they are based on normalized cross-correlation functions, lag maps of AMUA plumes capture differences in the timing of oscillation events across the electrode grid, but not their magnitudes. In a second analysis approach we therefore aimed to capture overall propagation directions of predominant concentrations of neural activity based on LFP. LFP fields are less localized than are action potential fields and, therefore, better reflect the magnitude of neural activity in our recordings. We identified, at 1-ms intervals, plumes of LFP in the electrode grid that were stronger (that is, more negative) than a threshold criterion of −250 mV, and tracked the changes of their spatial mean in time (see example, Figure 2G). Plumes propagate in variable trajectories within recordings, both in orthogonal horizontal and sagittal planes (Figure 5A). For each plume, we expressed the net movement across the plane of the electrode grid as a mean vector (Figure 5B). Group mean vectors per recording have significantly non-random directions (Rayleigh-tests; *P*<0.001) in every recording (*n* = 11), with an overall tendency for plumes in the hyperpallium to travel anteriorly, toward the surface of the brain (Figure 5C).

Our experimental animals were anesthetized, not sleeping spontaneously. In mammals, some kinds of anesthesia, including isoflurane, activate normal sleep promoting regions in the hypothalamus [29], induce slow oscillatory brain activity resembling that occurring during spontaneous NREM sleep [2,11], and are a well-established method for studying the intracerebral neurophysiological aspects of NREM sleep. Moreover, recent studies suggest that anesthesia may serve a functional role for the brain similar to that of sleep [37]. Importantly, mammalian slow-oscillations have been shown to travel both during anesthesia and NREM sleep [5,7-15]. For these collective reasons, we therefore think that the isoflurane-induced slow-oscillatory patterns of brain activity reported here are likely to be comparable to those of a freely sleeping animal. Indeed, the spectral power distribution recorded under isoflurane (Additional file 12: Figure S3) is similar to that recorded in the EEG during natural NREM sleep in zebra finches [33].

As in the laminar mammalian neocortex, slow-waves propagate through the nuclear avian brain. Thus, an important insight from our findings is that the propagating nature of slow-waves during NREM sleep and anesthesia does not reflect a neural phenomenon that is unique to mammals, or that depends upon uniquely mammalian cortical cytoarchitecture or computational properties. Rather, propagating slow-waves reflect a more general type of sleep-related neural activity. Propagating slow-waves may have evolved independently in birds and mammals, perhaps for functional reasons, despite their differing cortical cytoarchitectures, or it may represent an ancestral trait. Although visually evoked propagating two-dimensional waves have been described in the visual cortex of anesthetized turtles [38], it remains unclear whether the cortex of non-avian reptiles generate slow-oscillations during sleep [39].

Although the presence of traveling slow-waves in avian and mammalian brain structures is highly interesting from a comparative viewpoint, and may be linked in terms of function [20], we do not suggest they are necessarily identical phenomena. Three-hundred million years of separation is a long time even by evolutionary standards, and it would be surprising if this was not in any way reflected in the exact phenomenology of traveling slow-waves, especially given the known differences in cytoarchitecture. Nevertheless, we believe that, as in comparative work in general, differences may be as informative as similarities. Although the overall phenomenology and frequency content of EEG slow-waves during NREM sleep and anesthesia in birds and mammals appear similar, our recordings show that during apparent up-states there are often multiple sharp LFP peaks and AMUA bursts, each of which corresponds to a propagating plume. In mammals, each cycle of a traveling slow-wave (that is, an EEG peak) is thought to reflect one up-state, spreading from a site of origin to neighboring regions. Given the limited data on trans-membrane potentials during NREM sleep or anesthesia in birds, it is not possible to say whether multiple plumes arise and propagate during a single up-state or whether one propagating plume corresponds to one short up-state; although the former seems more likely given the single report of slow-oscillations in birds [19] and the duration of avian EEG slow-waves. Furthermore, it is difficult to conclude whether or not the situation of multiple-propagating-plumes-per-presumed-up-state that we find in birds is different from the one-traveling-wave-per-up-state situation described in mammals. High-frequency activity occurring during up-states in anesthetized cats is correlated across neurons spaced <0.3 mm apart [13], but poorly correlated across neurons spaced 4 mm apart. Although propagation of this fast activity between close neurons has not been examined to our knowledge, it is conceivable that such activity correlates and propagates on fine spatial scales similar to that examined here in birds. Additional studies in mammals and birds employing similar methods are needed to determine whether the propagation of higher frequency activity during apparent up-states is unique to birds.

While the propagating nature of slow-waves is shared by mammals and birds, the geometry of propagation differs between the two. In mammals, although slow-waves may engage different layers of the neocortex at slightly different times [18,40,41], propagation occurs primarily parallel to the layers of the neocortex. In contrast, in birds, slow-waves propagate as complex plumes of action and local field potential activity. While this difference is undoubtedly linked to the nuclear versus laminar arrangement of neurons in birds and mammals, respectively, it may also confer such neural cytoarchitecture with different computational properties.

Depending on the actual function of propagating slow-waves, the three-dimensional nature of plumes in the avian brain may have broader implications for understanding the evolution of the nuclear pallial structures subserving complex cognition in birds. While the propagating nature of slow-waves in general may simply be an epiphenomenon of excitable interconnected neurons [42], it may also play a role in processing information on a systems level. In the former case, while propagation *per se* may not serve a function, the slow-oscillation may be involved in processes occurring at the local level [3,7,20,43]. In the latter case, the propagating aspect of slow-waves may be involved in the successive reactivation and consolidation of recent memory traces [4], and/or their transfer and integration with older memories [1]. If correct, then the spatial dynamics of wave propagation may shape the nature of information processing [44]. In mammals, propagation occurs primarily parallel to the layers of the neocortex, restricting communication to two dimensions. In contrast, the nuclear arrangement of neurons in the avian brain and associated three-dimensional propagation of waves may confer greater degrees of computational freedom. Albeit speculative, such potential benefits may explain in part why birds 1) evolutionarily “replaced” the laminar dorsal cortex present in their reptilian ancestors with the nuclear hyperpallium, and 2) elaborated extensively upon pre-existing nuclear structures in the reptilian dorsal ventricular ridge to form the mesopallium and nidopallium, large nuclear structures that orchestrate complex cognitive abilities comparable to those orchestrated by high-order association regions in the laminar mammalian neocortex [26].

Finally, our findings may have implications for the interpretation of sleep-related neuronal activity in the song system of sleeping zebra finches, a prominent model for investigating sleep’s role in memory consolidation [45]. In anesthetized and spontaneously sleeping male finches, neurons in nuclei of the forebrain song system exhibit spontaneous bursts of activity [46,47]. Bursts in one nuclei often precede bursts in another distantly located nuclei, raising the possibility that this sequential activity is involved in processing song-related information [48,49]. As such, it would be interesting to determine whether this activity is part of larger plumes of activity propagating in the surrounding forebrain, or whether this activity propagates via dedicated pathways unaffected by surrounding plumes of activity.

## Conclusions

We demonstrate that slow-waves propagate as complex three-dimensional plumes of neuronal activity in the nuclear avian brain. These findings indicate that the propagating nature of slow-waves during NREM sleep and anesthesia does not reflect a neural phenomenon that is unique to mammals, or that depends upon uniquely mammalian cortical cytoarchitecture or computational properties. As such, efforts to understand the mechanisms responsible for propagating waves should not focus on lamination, *per se*, but rather on traits shared by mammalian and avian brains. In this regard, our findings refine our understanding of the neocortex. In addition, the three-dimensional nature of propagating plumes raises the intriguing possibility that such activity reflects computational processes that contributed to the evolution and elaboration of nuclear structures and associated complex cognition in birds. More generally, the presence of mammalian-like activity in the avian brain, despite differences in neuronal cytoarchitecture, suggests that the apparent absence of similar sleep-related activity in prominent invertebrate models of sleep, such as *Drosophila melanogaster*[50], cannot be simply attributed to their lack of a neocortex. Rather, such animals may lack neuronal properties (for example, slow-oscillations) involved in performing sleep functions shared by mammals and birds.

## Methods

### Animals

Thirteen adult (>90-day-old) female zebra finches were used in this study. The birds were reared in a colony and housed in an aviary with other adult zebra finches. All procedures were performed under systemic anesthesia under supervision of the institutional *Tierschutzbeauftragte*, in accordance with German laws and regulations (*Tierschutzgesetz, §4, Absatz 3 and §§8b, 9 Absatz 2, Satz 2*).

### Recordings

After the birds were anesthetized with isoflurane vaporized in oxygen (induction, 3%; maintenance, 1.0 to 1.25%), meloxicam (0.3 mg/kg; intramuscular injection) was administered for systemic analgesia. The head was fixed in a stereotaxic frame while the body rested on a heating pad to maintain body temperature. The angle of the head was 0 to 20º, except for one bird (Bird 13, caudomedial nidopallium, NCM) where it was 45º. After 20 minutes, the skull was exposed and a rectangular craniotomy was performed overlying the multi-electrode probe insertion point. An incision was made in the dura to allow for insertion of the probe. In most birds (*n* = 11) we recorded from the hyperpallium, 3.8 to 6.6 mm rostral, and centered around 0.9 to 2.3 mm lateral, from the bifurcation of the midsagittal sinus. In seven birds the probe was inserted horizontally, and in four birds sagittally. In six birds we recorded from the right hemisphere, and in five from the left hemisphere. Additionally, we recorded in two birds from within the caudal nidopallium (NC), a forebrain region that is maximally distant from the hyperpallium, one in the caudolateral nidopallium (NCL; 1.1 mm rostral, centered around 4.7 mm lateral) and the other one in the NCM (0.9 mm rostral, centered around 1.3 mm lateral).

To record neural activity, we used 64-channel silicon-based multi-electrode probes (NeuroNexus Technologies, Ann Arbor, MI, USA; a8 × 8 to 5 mm 200-200-413). These had eight parallel shanks (thickness: 15 μm, inter-shank spacing, 200 μm), each with eight iridium electrode sites (site surface, 413 μm^2^; inter-site spacing 200 μm), so that the 8 × 8 square matrix of electrode sites extended over an area 1,400 × 1,400 μm.

Initially, the tips of the probe shanks were positioned just above the brain surface to verify visually (magnification 60×) that the head was fixed well and did not move due to breathing or the heartbeat. The probe was then lowered in small steps to a position where the deepest shank reached its recording depth. In the hyperpallium, this was a depth of between 1,470 μm and 1,700 μm below the brain surface. This way, most electrode sites were situated in the hyperpallial nuclei (primarily the hyperpallium apicale, the most dorsal and thick nucleus), with the top-most sites just beneath the brain surface, while some of the deepest electrode sites were situated in the underlying mesopallium (Figure 1A). In the caudal nidopallium, recording depth was slightly deeper (2,400 μm in NCL and 1,920 μm in NCM). Recordings started 30 minutes after the probe was in place, and lasted 10 minutes in each bird.

Recorded signals were referenced to a silver wire under the scalp over the cerebellum and buffered by headstage preamplifiers (10× gain; MPA32I; MultiChannel Systems, Reutlingen, Germany) before amplification with a multichannel amplifier (250× gain; bandpass filters 0.1 to 5,000 Hz; PGA64; MultiChannel Systems, Austin, TX, USA). The amplified and filtered signals were digitized with a sampling frequency of 14 kHz and 16-bit resolution (NI9205; National Instruments), and stored on disk in HDF5 files [51].

At the end of the recording session, birds were sacrificed by increasing the level of isoflurane to 5%, after which the brain was removed and frozen for histology to determine electrode site placements [27].

### Data analyses

Analyses were performed using the scientific computing package SciPy, version 0.10 [52]. Recorded electrophysiological signals were filtered to high-frequency signals (0.5 to 5 kHz) containing action potentials, and low-frequency signals (0.1 to 350 Hz) containing LFP. The action potential signals were subsequently rectified so that their amplitude reflects the level of multiunit action potential firing in the vicinity of the electrode site (AMUA).

Correlation coefficients reported in the text were calculated using decimated (sampling rate 1.4 kHz) and normalized signals, and were based on full recordings. The correlation coefficient between two signals was defined as the absolute value of their cross-correlation function at *τ* = 0, that is, without a time lag. Mean between-site correlation coefficients are based on the mean of all possible site pairs (*n* = 64 × 63 = 4032). n-Values were in practice slightly lower because some sites were excluded (median 2 out of 64 sites per recording) because they were situated just above the brain surface or contained recording artifacts. Mean correlations between LFP and AMUA signals were based on the mean of all possible within-site cross-correlations.

AMUA plume propagation analyses (Figure 2E, F, Figure 4) were based on normalized cross-correlation functions, with time-lag, *τ*, ranging from −50 to +50 ms, in 1-ms time steps, for each identified plume (based on LFP signals, see below).

Temporospatial propagation of LFP plumes across the 8 × 8 recording electrode matrix was quantified using a different methodology (for example, Figure 2G). We identified a plume as a continuous episode with a duration of at least 20 ms where LFP levels are always lower than −0.25 mV at any three or more electrode sites. The number of plumes identified this way is dependent on the exact threshold that is chosen because weaker plumes may be missed, but using different thresholds (−0.20 mV and −0.30 mV) did not result in a qualitative change of the results reported here. The center of each plume (that is, its spatial mean) was determined at 1-ms intervals by calculating a weighted average of the spatial coordinates of sub-threshold sites (that is, <−0.25 mV), with their absolute potential as a weighting factor. The resulting temporal series of plume center locations is called a traveling plume “trajectory”. The median number of trajectories that were found in the 10-minute recordings was 697 (range: 232 to 969).

### Anatomy

Before implantation, the multi-electrode probes were coated with the fluorescent dye DiI for anatomical registration with histological sections. Frozen brains were cut into 25 μm sections using a freezing microtome and mounted on glass slides. Sections were fixed in 4% formaldehyde solution in phosphate-buffered saline, and then DAPI-stained. Fluorescence microscopy was used to verify probe location in 11 of 13 birds.

### Videos

Supplementary videos were rendered by low-pass filtering LFP and AMUA signals at 100 Hz and decimating them in the time domain with factors 560 (for real-time speed), 56 (for 0.1× speed), or 22 (for 0.04× speed) to obtain video-compatible sampling rates of 25 Hz.

## Abbreviations

AMUA: Analogue multiunit activity; EEG: Eectroencephalogram; LFP: Local field potential; NC: Caudal nidopallium; NCL: Caudolateral nidopallium; NCM: Caudomedial nidopallium; NREM: Non-rapid eye movement.

## Competing interests

The authors declare no competing financial interests.

## Author’ contributions

GJLB, JAL, and NCR designed the study. GJLB and JvdM collected the data. GJLB and NCR analyzed the data and wrote the paper. All authors read and approved the final manuscript.

## Supplementary Material

Additional file 1: Figure S1 Electrode sites with positive local field potential (LFP) peaks near the brain surface are not usually associated with strong analogue multiunit activity (AMUA) peaks in the same recording site, suggesting the absence of neurons with action potential firing at these sites. To quantitatively verify this observation we calculated for every site (*n* = 811 sites in 13 birds) the skewness of its LFP signal, which is negative for negatively peaked signals and positive for positively peaked signals, and the kurtosis of its AMUA signal, which is a measure for its peakedness. Signals with stronger action potential firing have higher kurtosis. A plot of AMUA kurtosis against LFP skewness shows that sites with negatively peaked LFP signals overall have much stronger action potential firing. The difference in kurtosis between sites with negatively peaked LFP (median kurtosis: 32.9; *n* = 716) and positively peaked LFP (median kurtosis: 2.3; *n* = 95) is highly significant (Mann–Whitney-U test; *u* = 11,657, *P* <0.001). Kurtosis is calculated as excess kurtosis, so that the kurtosis of a normal distribution equals zero. Click here for file

Additional file 2: Video S1 Video of the image sequence shown in Figure 2D. Click here for file

Additional file 3: Video S2 Video of the image sequence shown in Figure 3A. Click here for file

Additional file 4: Video S3 Video of the image sequence shown in Figure 3B. Click here for file

Additional file 5: Video S4 Video of the image sequence shown in Figure 3C. Click here for file

Additional file 6: Video S5 Video of the image sequence shown in Figure 3D. Click here for file

Additional file 7: Video S6 Longer example of temporospatial slow-wave patterns in Bird 5. The multi-electrode probe is situated in a horizontal plane in the hyperpallium (cf. Figure 1A). Click here for file

Additional file 8: Video S7 Longer example of temporospatial slow-wave patterns in Bird 9. The multi-electrode probe is situated in a horizontal plane in the hyperpallium (cf. Figure 1A). Click here for file

Additional file 9: Video S8 Longer example of temporospatial slow-wave patterns in Bird 1. The multi-electrode probe is situated in a sagittal plane in the hyperpallium (cf. Figure 1A). Click here for file

Additional file 10: Video S9 Longer example of temporospatial slow-wave patterns in Bird 12. The multi-electrode probe is situated in a horizontal plane in caudolateral nidopallium (NCL), a forebrain region that is almost maximally distant from the hyperpallium. Click here for file

Additional file 11: Figure S2 Distribution of the translation speed of local field potential (LFP) plumes centers across the 2-D electrode gird in Bird 5 (horizontal hyperpallial recording). Across birds, the mean speed ranged from 0.023 to 0.040 m/s (mean +/− SEM: 0.031+/− 0.002, *n* = 7 birds) in the horizontal plane, and from 0.021 to 0.027 m/s (mean +/− SEM: 0.024 +/− 0.002, *n* = 4 birds) in the sagittal plane. The speed differences between recordings from horizontal and sagittal planes are not statistically significant (*t* = 2.2, *p* = 0.06). The mean speed in the caudomedial nidopallium (NCM) (*n* = 1) and caudolateral nidopallium (NCL) (*n* = 1) recordings was 0.038 and 0.029, respectively. Plume speed was determined by calculating the LFP center (that is, spatial mean, see Methods) translation speed in 1-ms time steps (cf. Figure 2G), and taking the average per plume. Note that translation speed across the 2-D electrode grid does not necessarily correspond to the plume propagation speed through the brain, because plumes may have complex 3-D temporospatial dynamics and impinge upon the 2-D electrode grid at unknown angles. Click here for file

Additional file 12: Figure S3 Mean spectral power density of the local field potential (LFP) signals recorded in the hyperpallium shows a peak at 0.45 Hz. The mean (+/− SEM) was calculated over the power spectra from all hyperpallium recordings (*n* = 11 birds), selecting the same channel near the center of the electrode array. Power spectra were calculated using Welch’s method, using 10-s time windows and 99% overlap. Click here for file
