# Supplementary figures and images for: Plumes of neuronal activity propagate in three dimensions through the nuclear avian brain
Source: BMC Biol. 2014 Feb 28;12:16. doi: 10.1186/1741-7007-12-16 (PMC4015294; doi:10.1186/1741-7007-12-16)

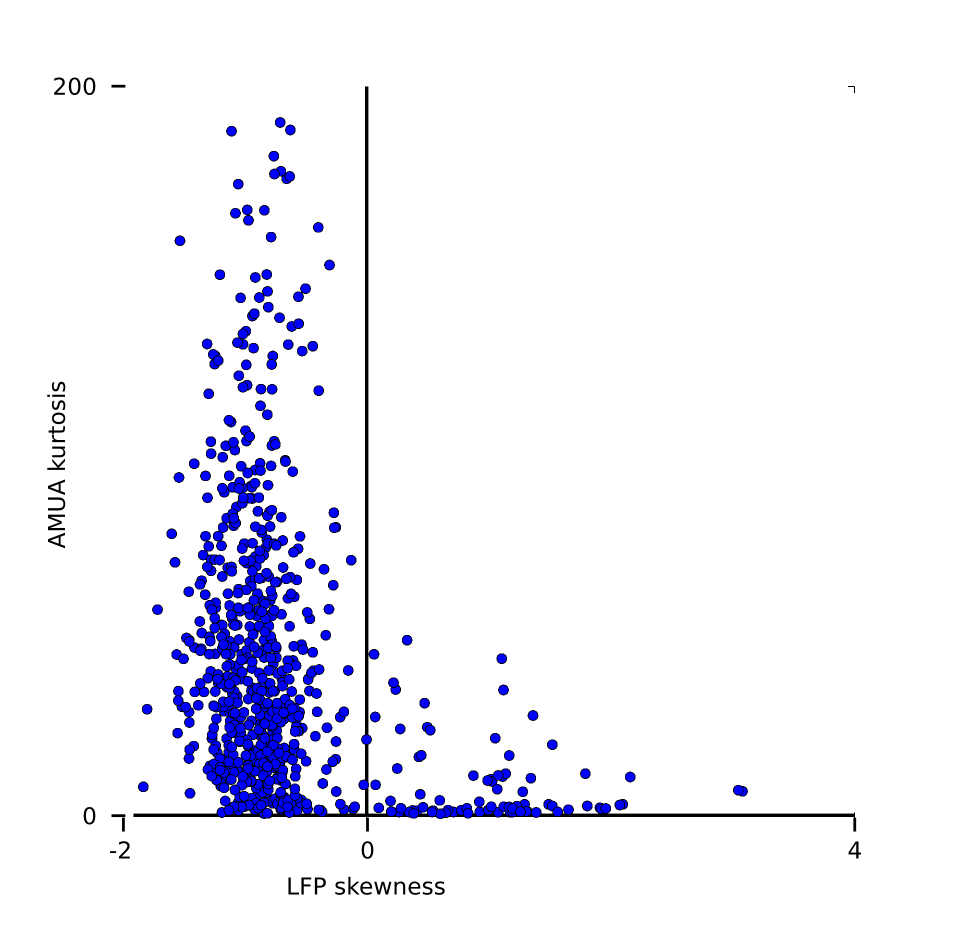

Supplement: Additional file 1: Figure S1 — Electrode sites with positive local field potential (LFP) peaks near the brain surface are not usually associated with strong analogue multiunit activity (AMUA) peaks in the same recording site, suggesting the absence of neurons with action potential firing at these sites. To quantitatively verify this observation we calculated for every site (n = 811 sites in 13 birds) the skewness of its LFP signal, which is negative for negatively peaked signals and positive for positively peaked signals, and the kurtosis of its AMUA signal, which is a measure for its peakedness. Signals with stronger action potential firing have higher kurtosis. A plot of AMUA kurtosis against LFP skewness shows that sites with negatively peaked LFP signals overall have much stronger action potential firing. The difference in kurtosis between sites with negatively peaked LFP (median kurtosis: 32.9; n = 716) and positively peaked LFP (median kurtosis: 2.3; n = 95) is highly significant (Mann–Whitney-U test; u = 11,657, P <0.001). Kurtosis is calculated as excess kurtosis, so that the kurtosis of a normal distribution equals zero. [file 1741-7007-12-16-S1.tiff]

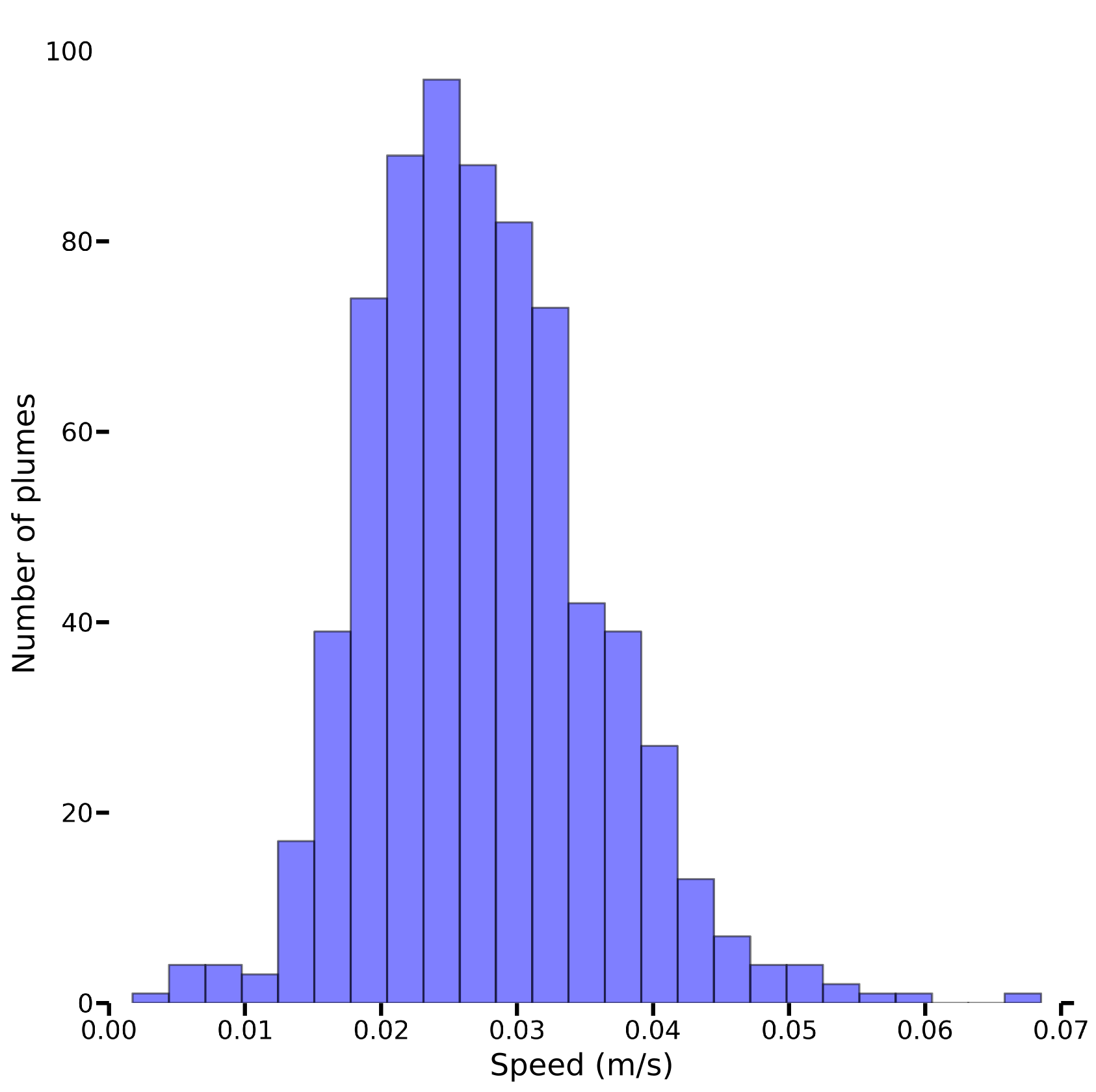

Supplement: Additional file 11: Figure S2 — Distribution of the translation speed of local field potential (LFP) plumes centers across the 2-D electrode gird in Bird 5 (horizontal hyperpallial recording). Across birds, the mean speed ranged from 0.023 to 0.040 m/s (mean +/− SEM: 0.031+/− 0.002, n = 7 birds) in the horizontal plane, and from 0.021 to 0.027 m/s (mean +/− SEM: 0.024 +/− 0.002, n = 4 birds) in the sagittal plane. The speed differences between recordings from horizontal and sagittal planes are not statistically significant (t = 2.2, p = 0.06). The mean speed in the caudomedial nidopallium (NCM) (n = 1) and caudolateral nidopallium (NCL) (n = 1) recordings was 0.038 and 0.029, respectively. Plume speed was determined by calculating the LFP center (that is, spatial mean, see Methods) translation speed in 1-ms time steps (cf. Figure 2G), and taking the average per plume. Note that translation speed across the 2-D electrode grid does not necessarily correspond to the plume propagation speed through the brain, because plumes may have complex 3-D temporospatial dynamics and impinge upon the 2-D electrode grid at unknown angles. [file 1741-7007-12-16-S11.tiff]

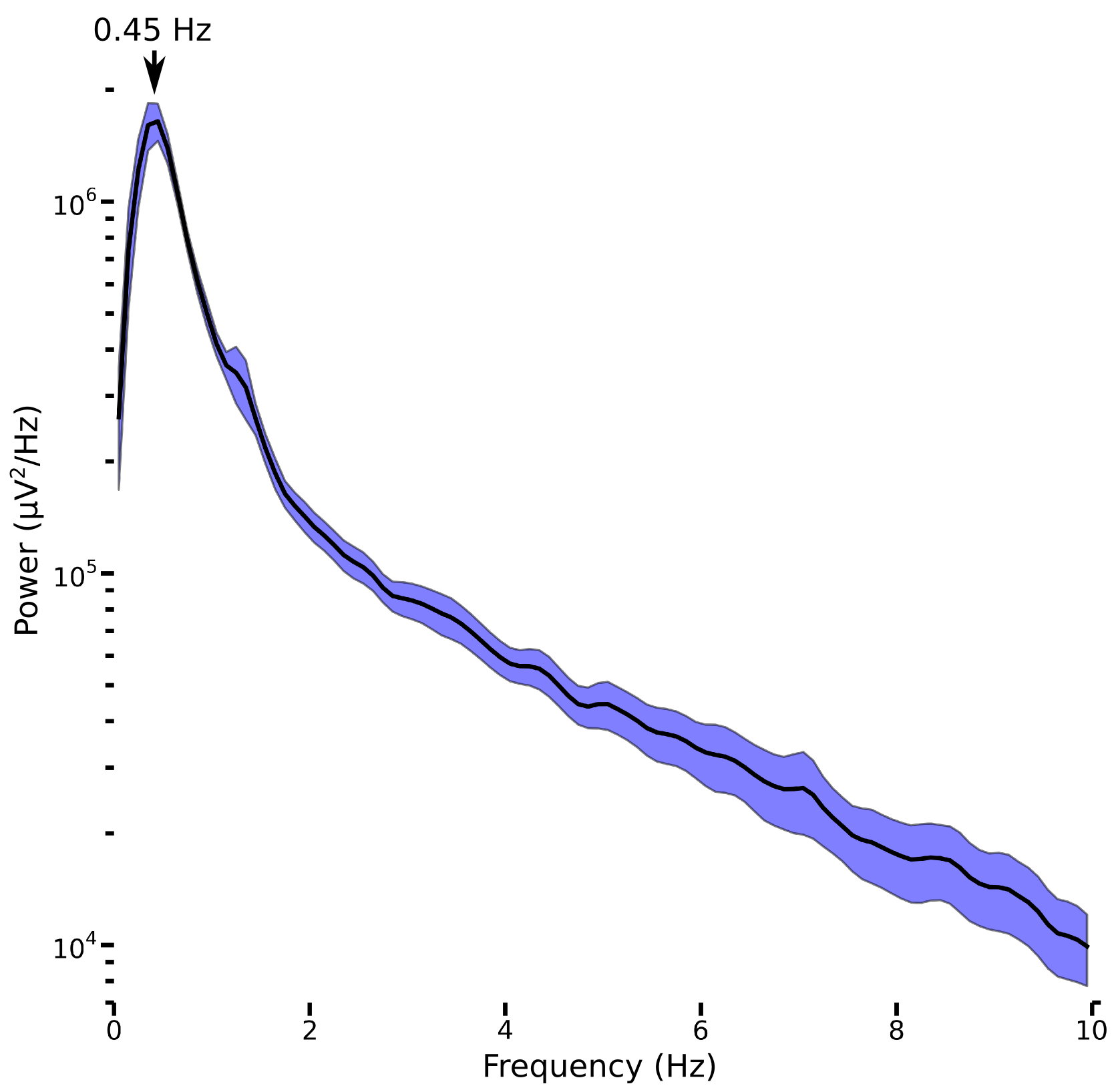

Supplement: Additional file 12: Figure S3 — Mean spectral power density of the local field potential (LFP) signals recorded in the hyperpallium shows a peak at 0.45 Hz. The mean (+/− SEM) was calculated over the power spectra from all hyperpallium recordings (n = 11 birds), selecting the same channel near the center of the electrode array. Power spectra were calculated using Welch’s method, using 10-s time windows and 99% overlap. [file 1741-7007-12-16-S12.tiff]
